# A prospective surveillance study for multidrug-resistant bacteria colonization in hospitalized patients at a Thai University Hospital

**DOI:** 10.1186/s13756-018-0393-2

**Published:** 2018-08-20

**Authors:** Pinyo Rattanaumpawan, Chatiros Choorat, Kanchanaporn Takonkitsakul, Teerawit Tangkoskul, Chakrapong Seenama, Visanu Thamlikitkul

**Affiliations:** 1 Division of Infectious Diseases and Tropical Medicine, Bangkok, Thailand; 2 Department of Medicine, Faculty of Medicine Siriraj Hospital, Mahidol University, 2 Wang Lang Rd., Bangkoknoi, Bangkok, 10700 Thailand

## Abstract

**Background:**

Colonization with multidrug-resistant (MDR) bacteria is a major risk factor for developing subsequent MDR infections.

**Methods:**

We performed a prospective surveillance study in hospitalized patients at Siriraj Hospital. Nasal cavity, throat, inguinal area and rectal swabs were obtained within the first 48-h after admission, on day-5 after hospitalization and then every 7 days until discharge. Target bacteria included extended-spectrum beta-lactamase-producing Enterobacteriaceae (ESBL), carbapenem-resistant-*P.aeruginosa* (CR-PA), carbapenem-resistant-*A.baumannii* (CR-AB) and methicillin-resistant *S.aureus* (MRSA).

**Results:**

From January 2013–December 2014, 487 patients were enrolled. The baseline prevalence of colonization by ESBL, CR-PA, CR-AB and MRSA at any site was 52.2%, 6.8%, 4.7% and 7.2%, respectively. After 3-week of hospitalization, the prevalence of colonization by ESBL, CR-PA, CR-AB and MRSA increased to 71.7%, 47.2%, 18.9% and 18.9%, respectively. Multivariable analysis revealed that diabetes mellitus and recent cephalosporin exposure were the independent risk factors for baseline colonization by ESBL. The independent risk factors for CR-AB and/or CR-PA colonization were cerebrovascular diseases, previous hospitalization, transfer from another hospital/a LTCF and previous nasogastric tube use, whereas those for MRSA colonization were previous fluoroquinolone exposure and previous nasogastric tube use.

**Conclusions:**

The baseline prevalence of colonization by ESBL was relatively high, whereas the baseline prevalence of colonization by CR-PA, CR-AB and MRSA was comparable to previous studies. There was an increasing trend in MDR bacteria colonization after hospitalization.

## Background

Antimicrobial resistance (AMR) is considered a major health threat. The consequences of multidrug-resistant (MDR) bacterial infections including high morbidity and mortality and economic loss have been well documented in many studies [1–3]. Colonization by MDR bacteria is considered a potential source of cross-transmission to other patients [4–6]. Moreover, colonization by MDR bacteria was found to be an independent risk factor for developing subsequent MDR bacterial infections in previous studies [5, 7].

The World Health Organization recognized AMR as a global health problem and recommended that Member States should strengthen the knowledge and evidence base through AMR surveillance and research in the global action plan on AMR [8]. Lack of AMR surveillance data contributes to underestimating the magnitude of AMR problem and halting the implementation of AMR control measures.

A surveillance study reported that the prevalence of rectal colonization by ESBL-producing Enterobacteriaceae among newly-hospitalized general medical patients in an Israel teaching hospital was only 8% [9]. After two weeks of hospitalization, the prevalence of colonization increased to 21% [9]. Surprisingly, the prevalence of rectal colonization by extended-spectrum beta-lactamase-producing Enterobacteriaceae in Thai community volunteers was remarkably high (32.0–66.5%) [10, 11].

Similar to ESBL-producing Enterobacteriaceae, the prevalence of *Pseudomonas aeruginosa* (PA) colonization varied across geographic locations. In a United States study, the prevalence of PA rectal colonization among intensive care unit (ICU) patients was 11.6% [12]. However, a recent Spanish study found that the prevalence of rectal colonization by non-drug resistant PA and extensive drug resistant PA in ICU patients was 27.0% and 4.0%, respectively [13].

Based on the data from a recent surveillance study performed in a medical ICU in Korea, active surveillance detected carbapenem-resistant-*Acinetobacter baumannii* (CR-AB) in 15.0% of patients, and approximately one-third of them later developed CR-AB infections [14]. Similar to the Korean study, the prevalence of CR-AB colonization in ICU patients at a US tertiary hospital was 13.5% [15].

Nasal colonization by methicillin-resistant *Staphylococcus aureus* (MRSA) has been widely investigated. The prevalence of MRSA nasal colonization varied from 4.1% in the US national surveillance in-patient data [16] to 9.0% among newly-hospitalized patients in an Israel teaching hospital [9]. Data on MRSA colonization at other sites in the body in addition to the nasal cavity is very limited.

Based on previous scientific evidence, the prevalence of MDR bacteria colonization varied across specific types of MDR bacteria, geographic regions and clinical settings [community, hospital or long-term care facility (LTCF)]. Although many studies have already investigated the prevalence of MDR bacteria colonization, most studies focused only on rectal or stool colonization by MDR gram-negative bacteria and nasal colonization by MRSA. Furthermore, these studies were not longitudinal studies that monitored changes in the prevalence of AMR bacteria colonization after hospitalization.

Given these considerations, we performed a prospective surveillance study for MDR bacteria colonization in hospitalized patients on admission and during hospitalization. The primary objective was to determine the prevalence of colonization by MDR bacteria in newly-hospitalized patients and the prevalence of new acquisition of MDR bacteria during hospitalization. The secondary objective was to identify risk factors for colonization by MDR bacteria and for new acquisition of MDR bacteria. Results from this study helped us determine the magnitude of AMR problem and the natural history of AMR colonization in hospitalized patients. Furthermore, the study could identify the patients at risk for MDR bacteria colonization who may subsequently develop infections due to these bacteria.

## Methods

### Study design and setting

During a 2-year study period (1 January 2013–31 December 2014), we performed a prospective surveillance study in eight general medical wards at Siriraj Hospital, which is a 2200-bed university hospital located in Bangkok, Thailand. The study protocol was approved by the Siriraj Institutional Review Board.

### Study population

The eligible subjects were all adults aged ≥18 years who had been hospitalized in general medical wards for less than 24 h. Subjects who were expected to be discharged or dead within 48 h or those with any contraindications for obtaining clinical specimens (i.e. a neutropenic patient (digital rectal examination or rectal swab culture was contraindicated), or having local infection at the site of surveillance culture) were excluded. Only subjects who agreed to participate and signed informed consent forms were enrolled.

### Microbiological surveillance of AMR bacteria

Clinical specimens from four sites including the nasal cavity, throat, skin at the inguinal area and rectum or stool were obtained from each patient within 48 h after hospitalization (time-1). Clinical specimens were subsequently obtained on day 5 ± 1 of hospitalization (time-2) and then every 7 days until the patient left the hospital (time-3, time-4 and so on). All clinical specimens were transferred in Stuart transport medium to the Laboratory of Division of Infectious Diseases, Department of Medicine.

The targeted MDR bacteria were ESBL-producing Enterobacteriaceae*,* CR-PA, CR-AB and MRSA. MacConkey agar supplemented with ceftriaxone for the isolation of MDR gram-negative bacteria and Mannitol Salt agar for the isolation of staphylococci were used for inoculating the clinical specimens collected from all sites.

Species identification and antimicrobial susceptibility tests were performed according to the performance standards for antimicrobial susceptibility testing recommended by the Clinical and Laboratory Standards Institute 2013 [17]. Species identification was performed using conventional biochemical tests. Identification of ESBL-producing bacteria was confirmed using the combination disc method. MRSA strains were determined using cefoxitin disc (30 mg) screening. Antimicrobial susceptibility testing was performed using the disc diffusion method.

Results of microbiology surveillance were directly reported to the study team and available (per request) for the service team (i.e. a responsible physician, an infectious disease consultant, etc.). However, there was no special infection control intervention for patients with colonization by target MDR-bacteria.

### Data collection

Medical records for the enrolled patients were reviewed for demographics, co-morbidities and clinical course. Data on any hospitalization, medication used, intervention and catheter use in the preceding 90 days prior to hospitalization were also obtained. Previous hospitalization included any stay at observation or emergency rooms for periodic monitoring and/or short-term treatment for longer than 24 h within 3 months prior to the index hospitalization.

### Statistical analysis

Categorical variables were summarized by frequency and proportion, whereas continuous variables were summarised by mean, median, standard deviation and range as appropriate. The prevalence of colonization by MDR bacteria was reported as percentage with a 95% confidence interval (95% CI). Wilcoxon-type test for trend analysis was performed to identify an increasing trend of colonization over time after hospitalization.

Multivariate logistic analysis was performed to identify the risk factors for colonization by MDR bacteria (at any site) and for new acquisition of MDR bacteria (at any site). A separate model was built for each MDR pathogen including 1) ESBL-producing Enterobacteriaceae; 2) CR-PA and/or CR-AB and 3) MRSA. Primary analysis was performed to compare cases with the specific MDR pathogens to controls without the given pathogen. Additionally, we performed a secondary analysis by comparing cases with the specific MDR pathogen to controls without any colonization.

Any associated variable with a *p*-value ≤0.20 was entered in a forward stepwise manner into the model. Any associated variables with a *p*-value < 0.20 was entered into the model. The likelihood ratio test was performed to confirm the model fit. For all calculations, a two-tailed *p*-value of < 0.05 was considered statistically significant. All calculations were performed using STATA version 14.0 (Stata Corp, College Station, TX).

## Results

### Baseline characteristics of patients

**Fig. 1:**
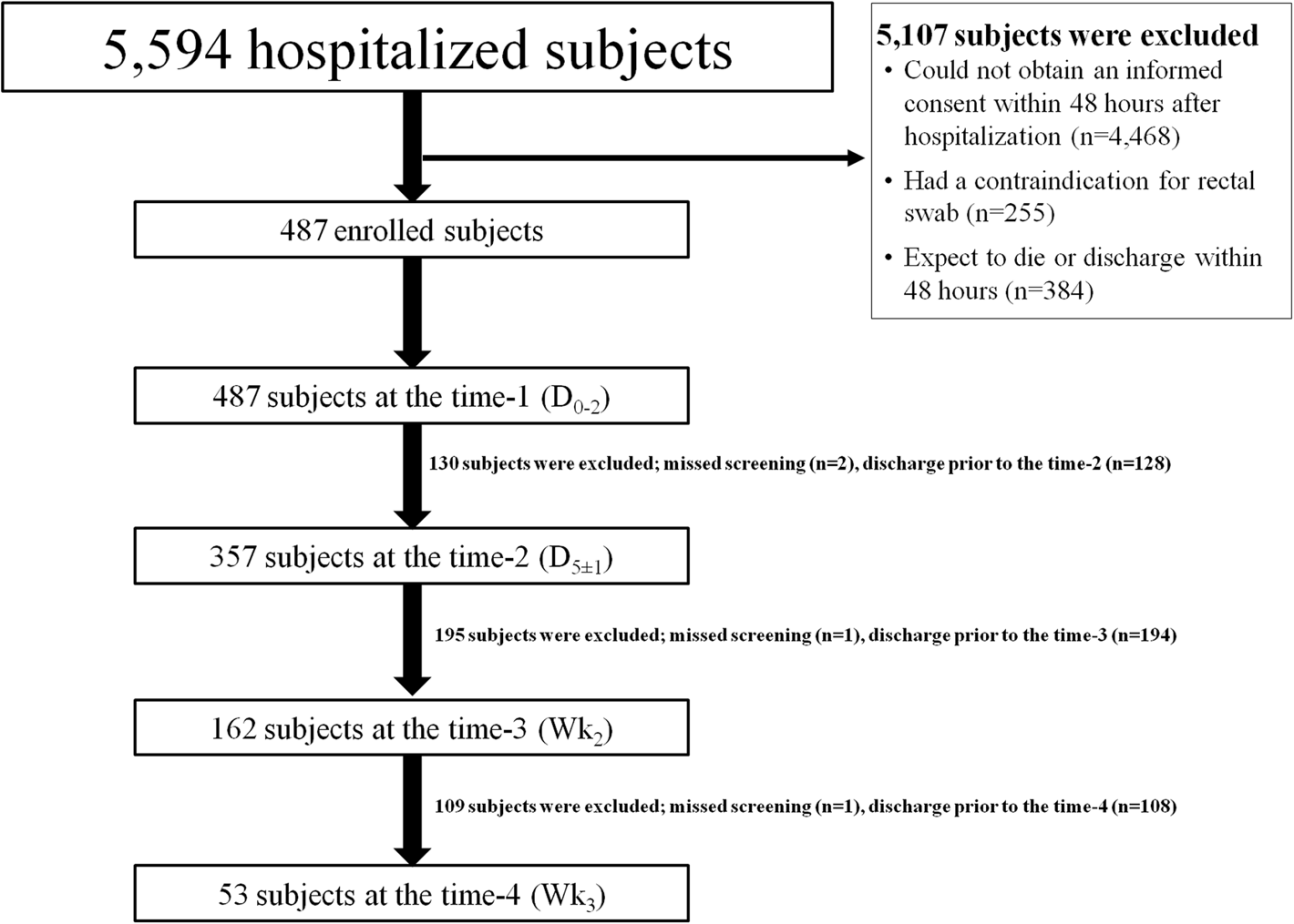
Study flow chart

During the study period, 487 patients were enrolled in the study as shown in Fig. 1. The baseline characteristics of patients prior to hospitalization are shown in Table 1. Nearly half (45.4%) of the patients were male, with an average age of 61.7 ± 17.8 years. Previous hospitalization was documented in 43.3% of patients. Additionally, 11.0% and 1.2% of patients had been transferred from another hospital or a LTCF, respectively. Majority of patients (94.4%) had at least one underlying disease.

**Table 1:** Baseline characteristics of 487 patients

Baseline characteristics prior to hospitalization	n (%)
Mean age ± SD (years)	61.7 ± 17.8
Male gender	221 (45.4%)
Mean length of hospital stay, days (±SD)	14.5 ± 18.5
Median length of hospital stay, days (range)	10.0 (2.0–303.0)
Previous hospitalization	211 (43.3%)
Transfer status	
From another hospital	56 (11.5%)
From a long-term care facility	6 (1.2%)
Underlying diseases	
Any underlying disease	420 (86.2%)
Hypertension	290 (59.6%)
Diabetes mellitus	184 (37.8%)
Cardiovascular diseases	133 (27.3%)
Cerebrovascular diseases	97 (19.9%)
Chronic liver diseases	88 (18.0%)
Chronic renal diseases	64 (13.1%)
Chronic lung diseases	63 (12.9%)
Malignancy	87 (17.9%)
Solid malignancy	75 (15.4%)
Hematologic malignancy	12 (2.5%)
Hematologic diseases	47 (9.7%)
Prior organ transplantation	7 (1.4%)
Receipt of any immunosuppressive agent within 90 days	75 (15.4%)
HIV infection	16 (3.3%)
Previous antibiotic exposure within 90 days after hospitalization	
Any antibiotic	148 (30.4%)
Penicillins	19 (3.9%)
Cephalosporins	60 (12.3%)
Carbapenems	33 (6.8%)
Beta-lactam/beta-lactamase inhibitors	27 (5.5%)
Fluoroquinolones	46 (9.4%)
Macrolides	19 (3.9%)
Others	42 (8.6%)
Previous use of indwelling catheters within 90 days	
Urinary catheter	73 (15.0%)
Nasogastric tube	45 (9.2%)

The most common underlying disease was hypertension (59.6%), followed by diabetes mellitus (DM) (37.8%) and cardiovascular disease (27.3%). One-third (30.4%) of patients had previously been exposed to at least one type of antibiotics within the past 3 months. Approximately 15% of patients had a long-term urinary catheter inserted prior to hospitalization. The mean length of stay (LOS) was 14.5 ± 18.5 days, whereas the median LOS was 10 (2–303) days.

### Colonization by MDR bacteria in newly-hospitalized patients

The prevalence of colonization by MDR bacteria in newly-hospitalized patients stratified by MDR bacteria species and by colonization site is shown in Table 2. Of the 487 patients evaluated, only 197 were free of colonization (40.5%). The rest (59.5%) were colonized by at least one specific MDR pathogen.

**Table 2:** Prevalence of colonization of MDR bacteria in newly-hospitalized patients (*N* = 487) stratified by the specific MDR bacteria and by the specimen collection site

MDR bacteria	All sites, n (%)	Nasal cavity, n (%)	Throat, n (%)	Inguinal area, n (%)	Rectum, n (%)
ESBL-producing Enterobacteriaceae	254 (52.2%)	13 (2.7%)	42 (8.6%)	80 (16.4%)	232 (47.6%)
*E. coli*	206 (42.3%)	5 (1.0%)	14 (2.9%)	58 (11.9%)	189 (38.8%)
*K. pneumoniae*	81 (16.6%)	9 (1.8%)	31 (6.4%)	29 (6.0%)	60 (12.3%)
Other Enterobacteriaceae	8 (1.6%)	0	1 (0.2%)	3 (0.6%)	7 (1.4%)
*A. baumannii*					
Carbapenem-susceptible	88 (18.0%)	13 (2.7%)	43 (8.8%)	34 (7.0%)	32 (6.6%)
Carbapenem-resistant	63 (12.9%)	13 (2.7%)	16 (3.3%)	32 (6.6%)	28 (5.8%)
*P. aeruginosa*					
Carbapenem-susceptible	33 (6.8%)	10 (2.1%)	11 (2.3%)	18 (3.7%)	12 (2.5%)
Carbapenem-resistant	23 (4.7%)	9 (1.9%)	18 (3.7%)	2 (0.4%)	6 (1.2%)
*Staphylococcus aureus*					
Methicillin-susceptible	49 (10.0%)	29 (6.0%)	21 (4.3%)	8 (1.6%)	11 (2.3%)
Methicillin-resistant	35 (7.2%)	21 (4.3%)	17 (3.5%)	6 (1.2%)	12 (2.5%)

More than half of the patients had ESBL-producing Enterobacteriaceae colonization in at least one body site, primarily in the rectum (47.6%) followed by the inguinal area (16.4%), throat (8.6%) and nasal cavity (2.7%). ESBL-producing *E. coli* (42.3%) were more prevalent than ESBL-producing *K. pneumoniae* (16.6%).

CR-PA was identified in only 4.7% of patients, primarily in the throat (3.7%). Baseline colonization by CR-AB was documented in 12.9% of patients, primarily in the inguinal area (6.6%) followed by the rectum (5.8%), throat (3.3%) and nasal cavity (2.7%).

MRSA was documented in 7.2% of patients, unlike MDR gram-negative bacteria, which primarily colonized in the nasal cavity (4.3%). The risk factors for baseline colonization by each MDR bacteria are reported in the next section.

### Colonization by MDR bacteria during hospitalization

**Fig. 2:**
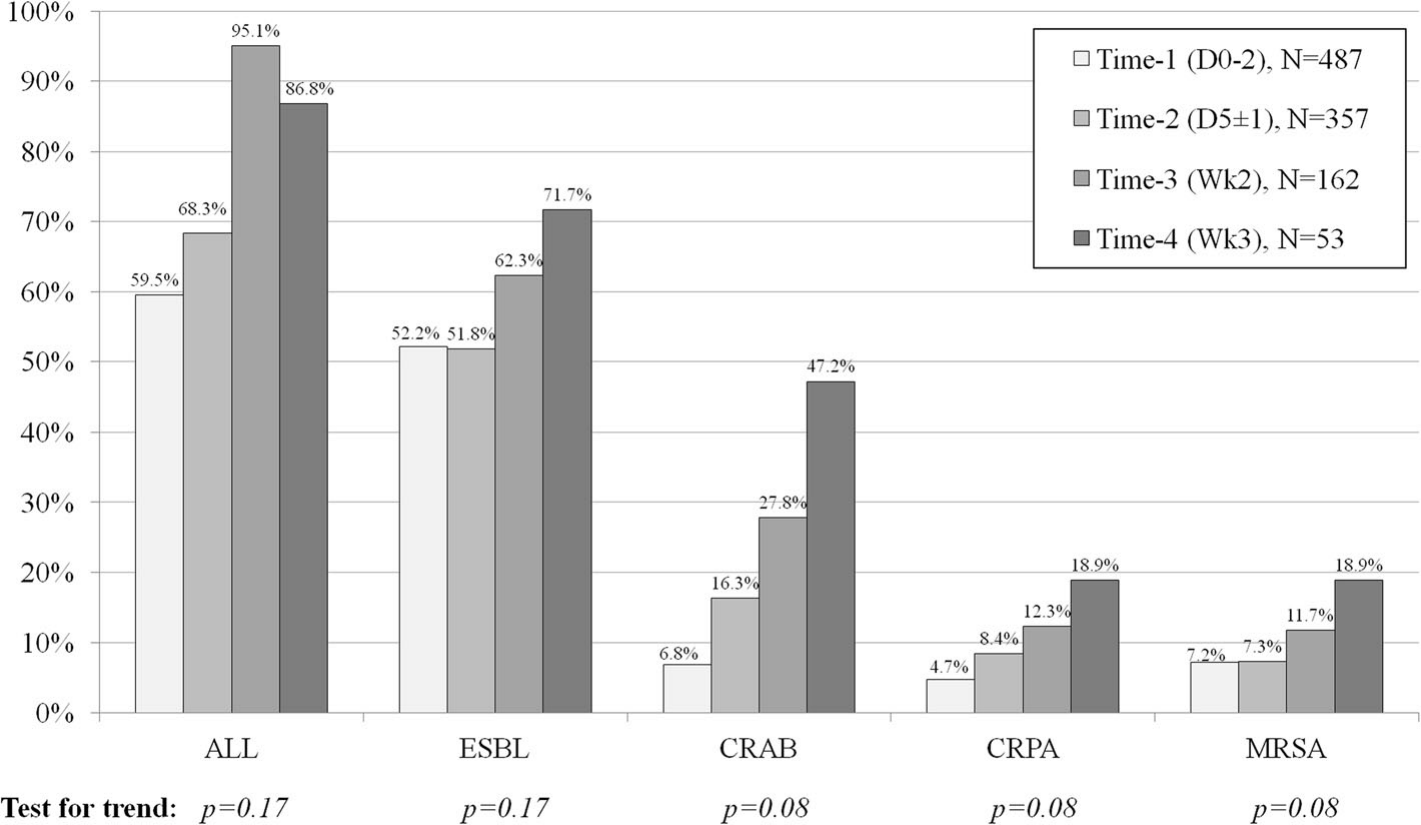
Prevalence of colonization by MDR bacteria stratified by the specific MDR bacteria and by the specimen collection time

Given that some patients were discharged or dead before subsequent clinical specimens were obtained, the number of follow-up specimens decreased over time. Collection of the subsequent specimens was successfully completed in 357 patients (73.3%) at time-2, 162 patients (33.3%) at time-3 and 53 patients (10.9%) at time-4. Overall colonization and colonization by all species of MDR bacteria showed an increasing trend over time as shown in Fig. 2. However, this increasing trend did not reach statistical significance in the test for trend analysis (all *p*-values > 0.05). The details of colonization by MDR bacteria stratified by MDR bacteria species, colonization site and specimen collection time are shown in Table 3. Due to the small number of new MDR bacteria acquisitions, we did not further investigate the risk factors for new acquisitions of these bacteria.

**Table 3:** Prevalence of colonization on admission and during hospitalization stratified by the specific MDR bacteria, the surveillance culture site and the time of specimen collection

Time	Any site, (%)	Nasal cavity, (%)	Throat, (%)	Inguinal area, (%)	Rectum, (%)
ESBL-producing Enterobacteriaceae					
Time-1 (*N* = 487)	52.2	2.7	9.8	17.5	50.4
Time-2 (*N* = 357)	51.8	3.4	15.1	17.5	46.2
Time-3 (*N* = 162)	62.3	8.3	25.0	21.9	46.2
Time-4 (*N* = 53)	71.7	12.0	28.6	27.1	53.8
ESBL-producing *E. coli*					
Time-1 (*N* = 487)	42.3	1.0	2.9	11.9	38.8
Time-2 (*N* = 357)	41.2	1.3	3.7	10.9	34.3
Time-3 (*N* = 162)	39.5	0.6	3.3	10.3	33.1
Time-4 (*N* = 53)	41.5	2.0	4.1	16.7	32.7
ESBL-producing *Klebsiella* spp.					
Time-1 (*N* = 487)	16.6	1.9	6.4	5.9	12.3
Time-2 (*N* = 357)	19.1	2.2	10.7	5.9	10.4
Time-3 (*N* = 162)	34.6	7.1	19.7	9.7	19.4
Time-4 (*N* = 53)	45.3	1.0	22.5	19.4	19.2
CR-AB					
Time-1 (*N* = 487)	6.8	2.1	2.3	3.7	2.5
Time-2 (*N* = 357)	16.3	4.7	6.7	10.4	8.6
Time-3 (*N* = 162)	27.8	8.3	13.2	15.5	10.0
Time-4 (*N* = 53)	47.2	26.0	24.5	22.9	19.2
CR-PA					
Time-1 (*N* = 487)	4.7	1.6	3.7	0.4	1.2
Time-2 (*N* = 357)	8.4	2.2	5.4	1.8	2.1
Time-3 (*N* = 162)	12.3	5.8	7.2	3.2	3.8
Time-4 (*N* = 53)	18.9	10.0	12.2	2.1	1.9
MRSA					
Time-1 (*N* = 487)	7.2	4.3	3.5	1.2	2.5
Time-2 (*N* = 357)	7.3	3.1	2.2	1.1	2.2
Time-3 (*N* = 162)	11.7	4.9	2.6	3.7	1.2
Time-4 (*N* = 53)	18.9	7.5	5.7	3.7	1.8

*Abbreviations: ESBL* Extended-Spectrum Beta-Lactamase, *CR-AB* Carbapenem-Resistant *Acinetobacter baumannii*, *CR-PA* Carbapenem-Resistant *Pseudomonas aeruginosa*, *MSSA* Methicillin-Susceptible *Staphylococcus aureus*, *MRSA* Methicillin-Resistant *Staphylococcus aureus*

### Risk factors for colonization by ESBL-producing Enterobacteriaceae in hospitalized patients

The risk factors for baseline colonization by ESBL-producing Enterobacteriaceae in 254 patients compared with 233 patients without ESBL-producing Enterobacteriaceae colonization are shown in Table 4. The independent risk factors from the primary multivariate analysis [Odds Ratio (OR); 95% CI; *p-value*] included underlying DM [1.45;1.00–2.10; *p = 0.05*] and previous exposure to cephalosporin [2.00;1.13–3.54; *p = 0.02*] as shown in Table 7. Secondary analysis identified similar risk factors with a similar OR as shown in the Table 7.

**Table 4:** Baseline characteristics prior to hospitalization for 254 patients (ESBL-producing Enterobacteriaceae) and 233 controls (no ESBL-producing Enterobacteriaceae)

Baseline characteristics	ESBL+ (*N* = 254)	ESBL- (*N* = 233)	*p*-value
Mean age ± SD (years)	61.4 ± 18.2	61.9 ± 17.4	0.76
Male gender	120 (47.2%)	101 (43.4%)	0.39
Previous hospitalization	115 (45.3%)	96 (41.2%)	0.37
Transfer status			
From another hospital	30 (11.8%)	26 (11.2%)	0.82
From a long-term care facility	4 (1.6%)	2 (0.9%)	0.47
Underlying diseases			
Any underlying disease	225 (88.6%)	195 (83.7%)	0.12
Hypertension	115 (61.0%)	135 (57.9%)	0.49
Diabetes mellitus	106 (41.7%)	78 (33.5%)	0.06
Cardiovascular disease	74 (29.1%)	59 (25.3%)	0.35
Cerebrovascular disease	56 (22.0%)	41 (17.6%)	0.22
Chronic liver disease	43 (16.9%)	45 (19.3%)	0.50
Chronic renal disease	37 (14.6%)	27 (11.6%)	0.33
Chronic lung disease	32 (12.6%)	41 (17.6%)	0.82
Malignancy	46 (18.1%)	41 (17.6%)	0.88
Solid malignancy	42 (16.5%)	33 (14.1%)	0.47
Hematologic malignancy	4 (1.6%)	8 (3.4%)	0.19
Hematologic diseases	26 (10.2%)	21 (9.0%)	0.65
Prior organ transplantation	2 (0.8%)	5 (2.2%)	0.21
Receipt of any immunosuppressive agent	33 (13.0%)	26 (11.2%)	0.54
HIV infection	10 (3.9%)	6 (2.6%)	0.40
Previous antibiotic exposure within 90 days after hospitalization			
Any antibiotic	84 (33.1%)	64 (27.5%)	0.18
Penicillins	12 (4.7%)	7 (3.0%)	0.33
Cephalosporins	40 (15.8%)	20 (8.6%)	0.02
Carbapenems	16 (6.3%)	17 (7.3%)	0.67
Beta-lactam/beta-lactamase inhibitors	10 (3.9%)	17 (7.3%)	0.11
Fluoroquinolones	26 (10.2%)	20 (8.6%)	0.53
Macrolides	11 (4.3%)	8 (3.4%)	0.61
Others	24 (9.5%)	18 (7.7%)	0.50
Previous use of indwelling catheters			
Urinary catheter	40 (15.8%)	33 (14.2%)	0.63
Nasogastric tube	26 (10.2%)	19 (8.2%)	0.43

### Risk factors for colonization by CR-PA and/or CR-AB in hospitalized patients

Due to the small number of cases with baseline colonization by CR-AB and CR-PA, we combined data on colonization by these two MDR bacteria. A total of 49 patients (10.1%) had at least one clinical specimen that grew CR-PA and/or CR-AB at the baseline. The risk factors for baseline colonization by CR-PA and/or CR-AB in 49 patients compared with 438 patients without this colonization are shown in Table 5. The independent risk factors identified in the primary analysis [OR; 95% CI; *p-value*] included previous hospitalization [2.21;1.07–4.53; *p = 0.03*], transfer from another hospital [2.67;1.19–5.98; *p = 0.02*] or a LTCF [11.51;1.84–71.83; *p = 0.01*], underlying cerebrovascular diseases [2.90;1.37–6.16; *p = 0.005*] and previous nasogastric tube use [2.38;1.002–5.67; *p = 0.05*]. Secondary analysis identified only three independent risk factors, specifically previous hospitalization, underlying cerebrovascular disease and previous nasogastric tube use, with slightly higher ORs. The results for both primary and secondary analyses are shown in Table 7.

**Table 5:** Baseline characteristics prior to hospitalization for 49 patients (with CR-AB and/or CR-PA) and 438 controls (without CR-AB and CR-PA)

Baseline characteristics	CR-AB and/or CR-PA (*n* = 49)	No CR-AB and CR-PA (*n* = 438)	*p*-value
Mean age ± SD (years)	66.7 ± 18	61.1 ± 17.8	0.04
Male gender	18 (36.7%)	203 (46.4%)	0.20
Previous hospitalization	32 (65.3%)	179 (40.9%)	0.001
Transfer status			
From other hospital	13 (26.5%)	43 (9.8%)	0.001
From a long-term care facility	4 (8.2%)	2 (0.5%)	< 0.001
Underlying diseases			
Any underlying disease	48 (98.0%)	372 (84.9%)	0.01
Hypertension	33 (67.4%)	257 (58.7%)	0.24
Diabetes mellitus	20 (40.8%)	164 (37.4%)	0.64
Cardiovascular disease	16 (32.7%)	117 (26.7%)	0.38
Cerebrovascular disease	23 (46.9%)	74 (16.9%)	< 0.001
Chronic liver disease	9 (18.4%)	79 (18.0%)	0.95
Chronic renal disease	8 (16.3%)	56 (12.8%)	0.49
Chronic lung disease	7 (14.3%)	56 (12.8%)	0.77
Malignancy	7 (14.3%)	80 (18.3%)	0.49
Solid malignancy	7 (14.3%)	68 (15.5%)	0.82
Hematologic malignancy	0	12 (2.7%)	0.24
Hematologic diseases	6 (12.2%)	41 (9.4%)	0.52
Prior organ transplantation	1 (2.0%)	6 (1.4%)	0.71
Receipt of any immunosuppressive agent	6 (12.2%)	- 53 (12.1%)	0.98
HIV infection	1 (2.0%)	15 (3.4%)	0.61
Previous antibiotic exposure within 90 days after hospitalization			
Any antibiotic	26 (53.1%)	122 (27.9%)	< 0.001
Penicillins	1 (2.0%)	18 (4.1%)	0.48
Cephalosporins	10 (20.4%)	50 (11.4%)	0.07
Carbapenems	9 (18.4%)	24 (5.5%)	0.001
Beta-lactam/beta-lactamase inhibitors	7 (14.3%)	20 (4.6%)	0.005
Fluoroquinolones	8 (16.3%)	38 (8.7%)	0.08
Macrolides	4 (8.2%)	15 (3.4%)	0.10
Others	8 (16.3%)	34 (7.8%)	0.04
Previous use of indwelling catheters			
Urinary catheter	19 (38.8%)	54 (12.3%)	< 0.001
Nasogastric tube	16 (32.7%)	29 (6.6%)	< 0.001

### Risk factors for colonization by MRSA in hospitalized patients

Of the 487 enrolled patients, 35 (7.2%) had at least one clinical specimen that grew MRSA at the baseline. Baseline characteristics for the 35 patients with MRSA colonization and 452 patients without MRSA colonization are shown in Table 6. Independent risk factors for baseline colonization by MRSA [OR; 95% CI; *p-value*] were previous fluoroquinolone exposure [2.76; 1.13–6.74; *p = 0.03*] and previous nasogastric tube use [6.60; 1.13–6.74; *p < 0.001*]. Stronger association between these two factors and baseline colonization by MRSA was documented in secondary analysis as shown in Table 7.

**Table 6:** Baseline characteristics prior to hospitalization for 35 patients (with MRSA) and 452 controls (without MRSA)

Baseline characteristics	MRSA (n = 35)	No MRSA (*n* = 452)	*p*-value
Mean age ± SD (years)	66.7 ± 20.1	61.3 ± 17.6	0.09
Male gender	22 (62.9%)	244 (54.0%)	0.31
Previous hospitalization	23 (65.7%)	188 (41.6%)	0.006
Transfer status			
From other hospital	5 (14.3%)	52 (11.3%)	0.59
From a long-term care facility	0	6 (1.3%)	1.00
Underlying diseases			
Any underlying disease	31 (88.6%)	389 (86.1%)	0.68
Hypertension	22 (62.9%)	268 (59.3%)	0.68
Diabetes mellitus	13 (37.1%)	172 (38.1%)	0.92
Cardiovascular disease	12 (34.3%)	121 (26.8%)	0.34
Cerebrovascular disease	15 (42.9%)	82 (18.1%)	< 0.001
Chronic liver disease	5 (14.3%)	83 (18.4%)	0.55
Chronic renal disease	3 (8.6%)	61 (13.4%)	0.60
Chronic lung disease	5 (14.3%)	58 (12.8%)	0.79
Malignancy	6 (17.1%)	81 (17.9%)	0.91
Solid malignancy	5 (14.3%)	70 (15.5%)	0.85
Hematologic malignancy	1 (2.9%)	11 (2.4%)	0.60
Hematologic diseases	3 (8.6%)	44 (9.7%)	1.00
Prior organ transplantation	0	7 (1.6%)	1.00
Receipt of any immunosuppressive agent	4 (11.4%)	55 (12.2%)	1.00
HIV infection	1 (2.9%)	15 (3.3%)	1.00
Previous antibiotic exposure within 90 days after hospitalization			
Any antibiotic	19 (54.3%)	129 (28.5%)	0.001
Penicillins	2 (5.7%)	17 (3.8%)	0.64
Cephalosporins	6 (17.1%)	54 (12.0%)	0.37
Carbapenems	6 (17.1%)	27 (6.0%)	0.01
Beta-lactam/beta-lactamase inhibitor	5 (14.3%)	22 (4.9%)	0.02
Fluoroquinolones	9 (25.7%)	37 (8.2%)	0.001
Macrolides	2 (5.7%)	17 (3.8%)	0.64
Others	5 (14.3%)	37 (8.2%)	0.22
Previous use of indwelling catheters			
Urinary catheter	13 (37.1%)	32 (7.1%)	< 0.001
Nasogastric tube	13 (37.1%)	60 (13.3%)	< 0.001

**Table 7:** Independent risk factors for baseline colonization by ESBL-producing Enterobacteriaceae, CR-AB and/or CR-PA and MRSA from the primary and secondary analyses

Variables	Adjusted OR [95% CI; *p-value*]	
	Primary Analysis (Case vs non-case)	Secondary Analysis (Case vs No MDR)
*1.* ESBL-producing *Enterobacteriaceae*	Case (*n* = 254) vs. non-case (*n* = 233)	Case (*n* = 254) vs. no MDR (*n* = 197)
Underlying diabetes mellitus	1.45 [1.00–2.10; *p = 0.05*]	1.49 [1.01–2.20; *p = 0.05*]
Previous cephalosporin exposure	2.00 [1.13–3.54; *p = 0.02*]	2.06 [1.11–3.81; *p = 0.02*]
*2.* CR-AB and/or CR-PA	Case (*n* = 49) vs. non-case (*n* = 438)	Case (*n* = 49) vs. no MDR (*n* = 197)
Previous hospitalization	2.21 [1.07–4.53; *p = 0.03*]	2.96 [1.40–6.26; *p = 0.004*]
Transfer from another hospital	2.67 [1.19–5.98; *p = 0.02*]	…
Transfer from a LTCF	11.51 [1.84–71.83; *p = 0.01*]	…
Underlying cerebrovascular disease	2.90 [1.37–6.16; *p = 0.005*]	2.68 [1.08–6.64; *p = 0.03*]
Previous nasogastric tube use	2.38 [1.002–5.67; *p = 0.05*]	4.13 [1.27–13.47; *p = 0.02*]
*3.* MRSA	Case (*n* = 35) vs. non-case (*n* = 452)	Case (*n* = 35) vs. no MDR (*n* = 197)
Previous fluoroquinolone exposure	2.76 [1.13–6.74; *p = 0.03*]	3.85 [1.26–11.80; *p = 0.02*]
Previous use of nasogastric tube	6.60 [1.13–6.74; *p < 0.001*]	12.86 [4.47–36.97; *p < 0.001*]

## Discussion

The present study revealed a remarkably high prevalence of baseline colonization by ESBL-producing Enterobacteriaceae compared with the prevalence from the Israel study (52.2% vs 8%) [9]. However, our baseline prevalence for faecal colonization by ESBL-producing Enterobacteriaceae (47.6%) was comparable with the prevalence of ESBL colonization among Thai community volunteers (32.0–66.5%) [10, 11].

Two important characteristics, namely DM and previous cephalosporin use, were identified as the independent risk factors for baseline colonization by ESBL-producing Enterobacteriaceae in this study. These findings were previously documented in many studies [9, 10]. Underlying DM may be a proxy for recurrent infections, previous antibiotic use and previous hospitalization [10, 18, 19]. Previous exposure to cephalosporin would result in selective pressure against non-ESBL-producing pathogens to become resistant to cephalosporin, leading to colonization in the patients [20].

This study revealed the comparable prevalence of CR-PA colonization (4.7%) compared with the results from the Spanish ICU study (4.0%) [13]. Additionally, the prevalence of CR-AB colonization (12.9%) was similar to the findings from previous studies performed in ICU patients (13.5–15.0%) [14, 15]. Although our study included only hospitalized patients in general medical wards, these patients were sicker than those hospitalized in a general medical ward in developed countries due to resource limitations. These statements could be confirmed due to a very high proportion of patients with co-morbidities (> 80%). Furthermore, approximately 40% of our enrolled patients had been previously hospitalized and more than 30% had a previous history of antibiotic exposure. These factors may explain the comparative prevalence of CR-PA and CR-AB colonization.

The independent risk factors for CR-AB and/or CR-PA colonization identified in this study were underlying cerebrovascular disease (CVA), previous hospitalization, transfer from another hospital or a LTCF and previous nasogastric tube use. Previous hospitalization and transfer from another hospital or a LTCF are well known risk factors for colonization by MDR bacteria. Neurologic disease was previously documented as an independent risk factor for PA colonization [12]. Furthermore, underlying CVA may be a proxy of aspiration pneumonia, previous nasogastric tube use, functional disability and previous hospitalization [21].

Our results for the baseline prevalence of MRSA colonization (7.2%) were comparable with results from previous studies [9, 16]. Significant risk factors for MRSA colonization identified in our study included previous fluoroquinolone exposure and previous nasogastric tube use. Previous fluoroquinolone exposure is well documented as an independent risk factor for MRSA colonization in many observational studies [22, 23]. Recent use of nasogastric tube was previously identified to be a significant risk factor for MRSA nasal colonization in end-stage renal disease patients [24].

The present study had several strengths. It was specifically designed to determine the prevalence of MDR bacteria colonization at various sites (nasal cavity, throat, skin at the inguinal area and rectum) and by a variety of important MDR bacteria (ESBL-producing Enterobacteriaceae, CR-PA, CR-AB and MRSA). Additionally, clinical specimens were collected at various time points to capture additional acquisition rates of colonization by MDR bacteria after hospitalization. Furthermore, we thoroughly collected all clinical characteristics that may be associated with baseline colonization by MDR bacteria.

The present study had some limitations. First, there was a small number of follow-up cultures, with only 53 specimens collected at time-4. Given that sicker patients are more likely to have a longer LOS with more collected clinical specimens, the prevalence of colonization after hospitalization may not represent the true prevalence. Second, the study results may be applicable to only tertiary care university hospitals. As we mentioned before, patients in our study were relatively sicker than those hospitalized at a general medical ward in developed countries.

## Conclusion

The prevalence of baseline colonization by ESBL-producing Enterobacteriaceae was relatively high, whereas the prevalence of baseline colonization by CR-PA, CR-AB and MRSA was comparable with the results from previous studies in other geographical locations. There was a slightly increasing trend of MDR bacteria colonization by all important pathogens after hospitalization. However, these observations did not reach statistical significance. Previous antibiotic use and previous nasogastric tube use were the common risk factors for various species of MDR pathogens. The documented risk factors from our study may be used to identify patients who are at a risk for MDR bacterial infection. A study with a larger sample size would be needed to identify the risk factors for acquiring new MDR colonization after hospitalization. Measures to prevent or delay colonization by MDR bacteria in hospitalized patients should be employed.

## Abbreviations

AMR: Antimicrobial resistance
CR-AB: Carbapenem-resistant-*A. baumannii*
CR-PA: Carbapenem-resistant-*P. aeruginosa*
CVA: Cerebrovascular disease
DM: Diabetes mellitus
ESBL: Extended-spectrum beta-lactamase-producing
HIV: Human immunodeficiency virus
ICU: Intensive care unit
LTCF: Long-term care facility
MDR: Multidrug-resistant
SD: Standard deviation
